# Viral Preservation with Protein-Supplemented Nebulizing Media in Aerosols

**DOI:** 10.1128/aem.01545-22

**Published:** 2023-03-01

**Authors:** Brittany Humphrey, Matthew Tezak, Mia Lobitz, Anastasia Hendricks, Andres Sanchez, Jake Zenker, Steven Storch, Ryan D. Davis, Bryce Ricken, Jesse Cahill

**Affiliations:** a Sandia National Laboratories, Albuquerque, New Mexico, USA; University of Nebraska-Lincoln

**Keywords:** MS2, phiX174, nebulizer, impinger, AGI, BSA, phi6, surrogate

## Abstract

The outbreak of SARS-CoV-2 has emphasized the need for a deeper understanding of infectivity, spread, and treatment of airborne viruses. Bacteriophages (phages) serve as ideal surrogates for respiratory pathogenic viruses thanks to their high tractability and the structural similarities tailless phages bear to viral pathogens. However, the aerosolization of enveloped SARS-CoV-2 surrogate phi6 usually results in a >3-log_10_ reduction in viability, limiting its usefulness as a surrogate for aerosolized coronavirus in “real world” contexts, such as a sneeze or cough. Recent work has shown that saliva or artificial saliva greatly improves the stability of viruses in aerosols and microdroplets relative to standard dilution/storage buffers like suspension medium (SM) buffer. These findings led us to investigate whether we could formulate media that preserves the viability of phi6 and other phages in artificially derived aerosols. Results indicate that SM buffer supplemented with bovine serum albumin (BSA) significantly improves the recovery of airborne phi6, MS2, and 80α and outperforms commercially formulated artificial saliva. Particle sizing and acoustic particle trapping data indicate that BSA supplementation dose-dependently improves viral survivability by reducing the extent of particle evaporation. These data suggest that our viral preservation medium may facilitate a lower-cost alternative to artificial saliva for future applied aerobiology studies.

**IMPORTANCE** We have identified common and inexpensive lab reagents that confer increased aerosol survivability on phi6 and other phages. Our results suggest that soluble protein is a key protective component in nebulizing medium. Protein supplementation likely reduces exposure of the phage to the air-water interface by reducing the extent of particle evaporation. These findings will be useful for applications in which researchers wish to improve the survivability of these (and likely other) aerosolized viruses to better approximate highly transmissible airborne viruses like SARS-CoV-2.

## INTRODUCTION

The question of how respiratory viruses are spread has been the subject of intense research for several decades (1, 2). Despite our progress, the SARS-CoV-2 pandemic has illustrated that there are still many unanswered questions, e.g., after aerosolization, how long do viable viruses persist in the air and what factors contribute to their persistence and spread? Large aerosol particles gravitationally settle in minutes in a room after coughing or sneezing (3). Importantly, from a clinical perspective, particles larger than ~5 μm do not typically deposit in the alveoli while particles up to tens of micrometers deposit in intrathoracic conducting airways and larger aerosols are trapped in the nose and mouth during inhalation (4). Fine aerosol mists do not follow a simple ballistic trajectory and can stay airborne for hours (2, 3). Exposure of respiratory particles to air leads to evaporation of water. This evaporation period has a complex relationship with viral viability, with some studies suggesting that desiccation can lead to viral preservation at long timescales (5) while another suggests that rapid desiccation can reduce viability (6). Therefore, the extent to which fine aerosols retain infectious virus particles continues to be debated (2, 7). The answers to these questions should directly impact public policy and the development of materials or technologies designed to inactivate, capture, or contain airborne viral threats.

Phages, the viruses of bacteria, offer a promising model to study these questions since many key structural features of phages closely approximate those of viral pathogens. Using phages as surrogates for pathogenic viruses allows investigations to proceed at the lowest biosafety level. Furthermore, the high growth rate of bacteria means that the number of viable phage particles can be quantified much faster than that of their eukaryote virus counterparts (8, 9). Indeed, tailless phages like phi6, MS2, and phiX174 are commonly used surrogates for pathogenic respiratory viruses (10). phi6 is generally considered a top surrogate for coronaviruses (CoVs), sharing many key features, including an RNA genome, similar size, a lipid envelope, and spike proteins (11). However, studies have shown that the number of viable phi6 viruses significantly decreases with time when exposed to ambient air conditions in aerosols or droplets (12, 13). This limitation makes phi6 a poor surrogate for studies focused on the spread, infectivity, or containment of airborne coronaviruses.

A recent study examined the viability of phi6 in droplets after deposition on glass by spray bottles. The authors showed that phi6 suspensions prepared in human saliva prior to aerosolization had significantly improved viability relative to phages that were prepared in standard phage storage buffer (suspension medium [SM] buffer) or water (13). Although the exact reason is not known, saliva is protective to viruses in aerosols and there have been recent efforts to develop nebulizing medium that reproduces the protective effect of saliva; however, these studies have shown mixed results (14) or focus on characterizing a single surrogate virus (15). These reports motivated us to identify common and inexpensive lab reagents that could be used in nebulizing medium designed to recapitulate the protective effect that saliva confers on airborne viruses. We employed a simple aerosol testbed to compare different nebulizer media and the effects on the viability of four distinct classes of phages. These phages include phi6 (double-stranded RNA [dsRNA] genome, lipid envelope), MS2 (single-stranded RNA [ssRNA] genome, protein capsid), phiX174 (single-stranded DNA [ssDNA] genome, protein capsid), and 80α (tailed phage, double-stranded DNA [dsDNA] genome, protein capsid).

## RESULTS

### A viral preservation medium: BSA confers protection on airborne viruses.

**FIG 1:**
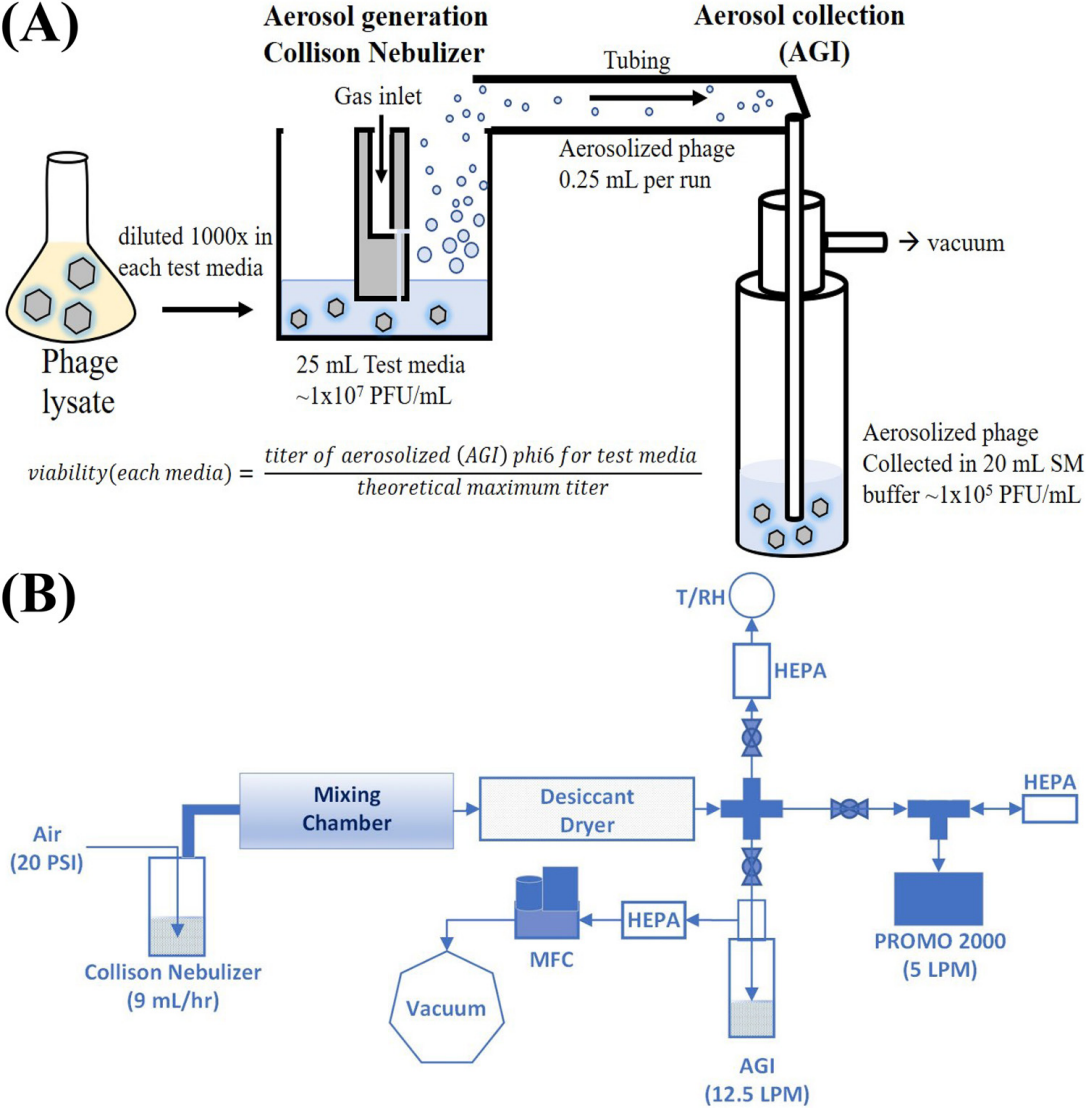
A cartoon (A) and diagram (B) describing the biological aerosol exposure testbed. MFC, mass flow controller. For each spray, the nebulizing medium in the Collison nebulizer was replaced and collected samples were withdrawn from the AGI.

**FIG 2:**
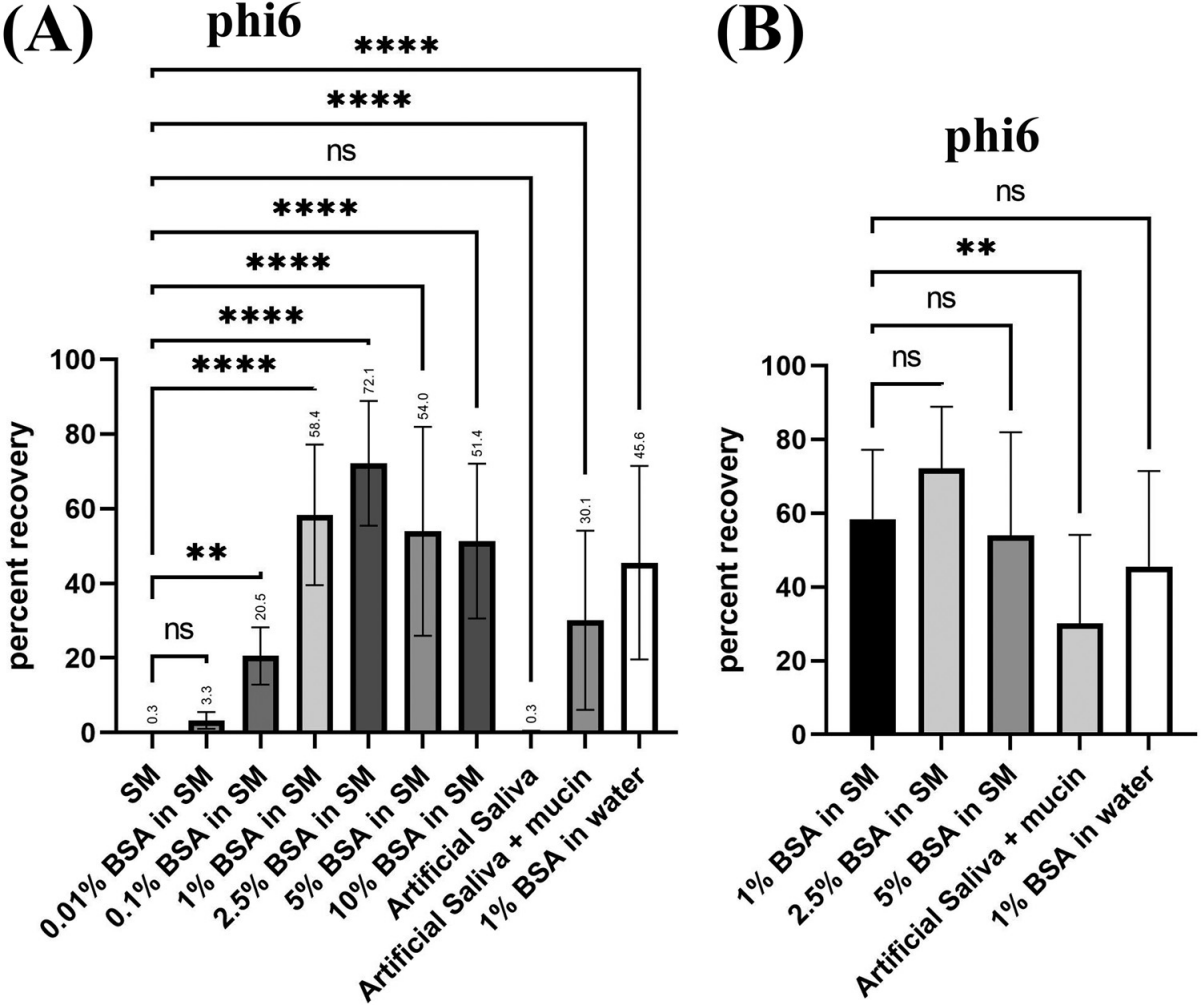
The percent recovery of phi6 aerosolized in SM/BSA, water/BSA, or artificial saliva with/without mucin. (A) phi6 at ~1 × 10^7^ PFU/mL was prepared in SM buffer supplemented with the indicated concentrations of BSA, artificial saliva with or without mucin, or 1% BSA in water. (B) Comparison of 1% BSA in SM to other high-recovery media. For each set, 0.25 mL of phi6 in the indicated buffers was aerosolized using a Collison nebulizer. Aerosols were recovered in an all-glass impinger filled with 20 mL SM buffer. Averages from three technical replicates for six biological replicates are shown. Error bars represent standard deviation. Significance determined by one way analysis of variance and denoted as follows: ns (not significant), *P* > 0.05; *, *P* ≤ 0.05; **, *P* ≤ 0.01; ***, *P* ≤ 0.001; ****, *P* ≤ 0.0001.

Recent work demonstrated that the survival of phi6 and MS2 was improved when microliter-sized droplets were supplemented with bovine serum albumin (BSA) (16). Similarly, aerosol-focused studies have observed protective effects for viruses aerosolized in allantoic fluid (10) and protein-rich medium (14, 15). This led us to wonder whether total dissolved protein itself may be generally protective of viruses in aerosols. To address this question, we supplemented SM buffer with different concentrations of BSA to compare how different amounts of total protein in nebulizing solution affect the viability of airborne viruses. Experimental nebulizing solutions were prepared by diluting phi6 from a high-titer lysate (~1 × 10^10^ PFU/mL) 1,000-fold. The solutions were aerosolized in our aerosol testbed as described in Materials and Methods (Fig. 1). Percent recovery is the ratio of the titer recovered after aerosolization over the theoretical maximum titer (see Materials and Methods). We detected a nearly 3-log_10_ loss in recovery for phi6 aerosolized in SM buffer (Fig. 2). The reduction may be attributed to a combination of physical losses (e.g., losses due to attraction between droplets and the aerosol system’s components) and decay in viral viability. The magnitude of these losses is consistent with what others have shown when phi6 is exposed to ambient air conditions in aerosols or droplets (13, 17).

As we hypothesized, supplementation of BSA into the nebulizing solution resulted in significant improvement in the number of viable phi6 phage recovered after aerosolization (Fig. 2). The highest average increase was observed for 2.5% BSA, resulting in an ~240-fold improvement in recovery relative to SM buffer. However, above 1% BSA, average increases in recovery were not statistically significant (Fig. 2B). Likewise, no significant change was detected when we compared equivalent concentrations of BSA dissolved in SM buffer to water. Notably, the buffered solution showed less variability, likely due to the buffer providing a stabilizing effect on BSA. The 1% BSA/SM medium outperformed a commercially formulated artificial saliva medium containing mucin (Fig. 2B); however, there was no difference between SM buffer and artificial saliva formulations that lacked the mucin supplement.

**FIG 3:**
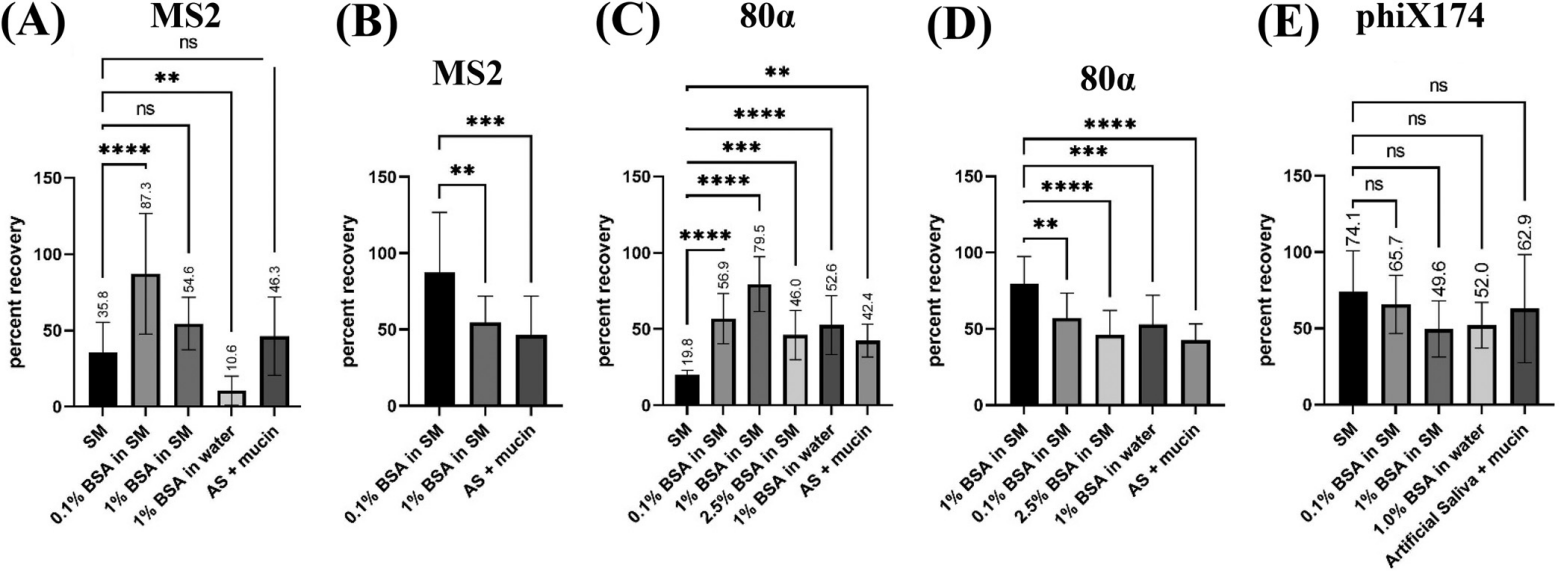
The percent recovery of MS2, 80α, and phiX174 aerosolized in SM/BSA, water/BSA, or artificial saliva with/without mucin. MS2 (A and B), 80α (C and D), and phiX174 (E) phages were prepared in SM buffer supplemented with the indicated concentrations of BSA. (B) Comparison of 0.1% BSA in SM to other high-recovery media for MS2. (D) Comparison of 1% BSA in SM to other high-recovery media for 80α. For each set, 0.25 mL of phage in the indicated buffers was aerosolized using a Collison nebulizer. Aerosols were recovered in an all-glass impinger filled with 20 mL SM buffer. Averages from three technical replicates for six biological replicates are shown. Error bars represent standard deviation. Significance determined by one-way analysis of variance and denoted as follows: ns (not significant), *P* > 0.05; *, *P* ≤ 0.05; **, *P* ≤ 0.01; ***, *P* ≤ 0.001; ****, *P* ≤ 0.0001.

Next, we tested whether BSA/SM medium was protective for other phages given the same aerosol treatment using downselected nebulizing medium formulations. We selected two other tailless phages commonly used in aerobiology as surrogates for pathogenic viruses (MS2 and phiX174). We also tested tailed Staphylococcus aureus phage 80α to investigate whether the protective effect extends beyond the standard set of surrogates. The recovery of MS2, 80α, and phiX174 in our baseline medium (SM buffer) alone was significantly higher than that of phi6 (36%, 20%, and 74%, respectively, compared to 0.3% for phi6). Like phi6, BSA supplementation in SM buffer improved the recovery of MS2 and 80α (Fig. 3) by ~2.4- and 4-fold relative to BSA-free SM, respectively. The best-performing BSA/SM formulation for MS2 (0.1%) contained significantly less BSA than 80α (1%) and phi6 (~2.5%). As with phi6, we identified BSA concentrations that outperformed mucin-supplemented artificial saliva for both MS2 and 80α (Fig. 3B and D). Interestingly, 1% BSA in SM buffer showed significantly higher recovery than 1% BSA in water for MS2 and 80α but not phi6. A simple explanation for the improvements in the SM buffer background is that the components in SM buffer affect the conformational stability of BSA and/or its behavior at the air-water interface (18). However, it is not clear why these improvements are phage dependent. For phiX174, there was no significant difference in recovery for any of the formulations tested (Fig. 3). phiX174 showed the highest recovery (74%) in our baseline nebulizing medium, suggesting that this phage is inherently stable against the stresses of aerosolization produced by our testbed. Indeed, the stability of phiX174 has been compared to that of pathogens like parvovirus that are highly resistant to environmental challenges (19). The finding that each nebulizing medium shows approximately the same recovery for phiX174 serves as a control for our experiments, demonstrating that the improvement in recoveries for the other phages is not simply due to differences in the mass transfer of viruses from nebulizer to collector.

### Average particle sizing: similar trends with small increases in droplet size.

**FIG 4:**
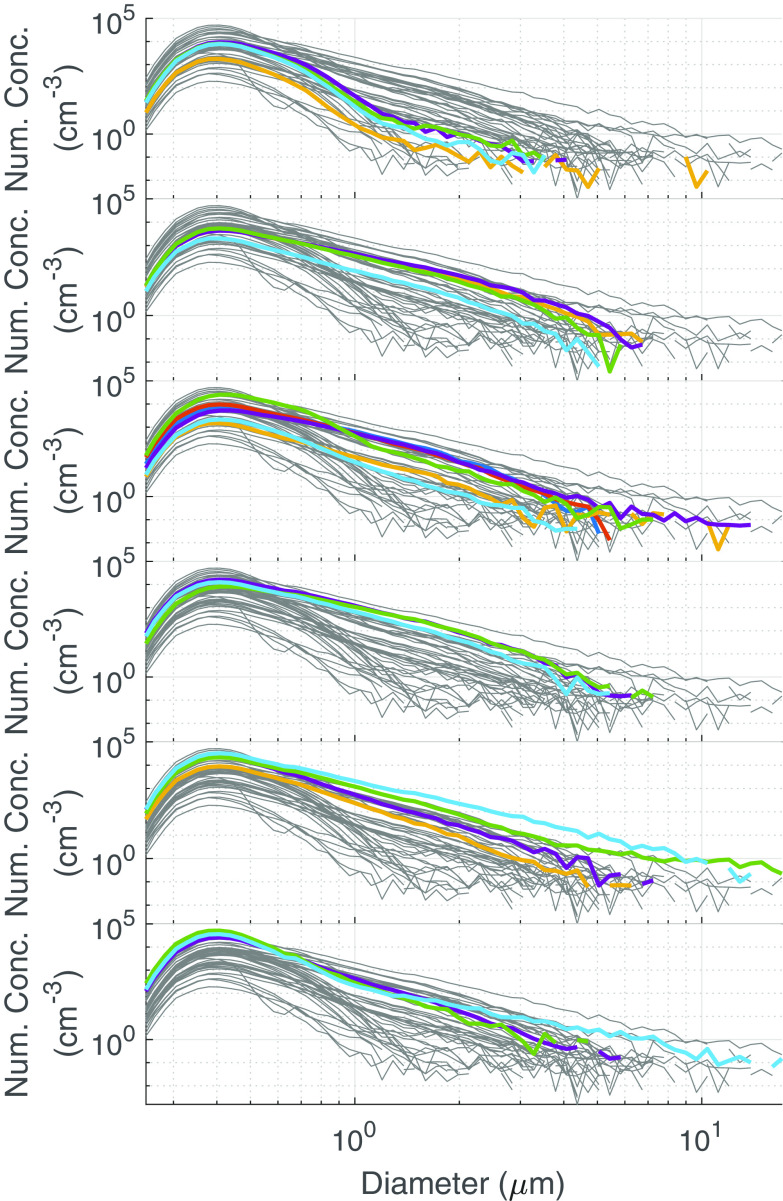
Average particle size distribution in number concentration. Data were obtained from a Welas Promo 2000 high-resolution aerosol spectrometer. The gray traces correspond to all data aligned on the same graph for reference. The colored traces are replicates corresponding to the indicated BSA concentration in each panel. The panels from top to bottom are: 0.01% BSA, 0.1% BSA, 1% BSA, 2.5% BSA, 5% BSA, and 10% BSA.

**FIG 5:**
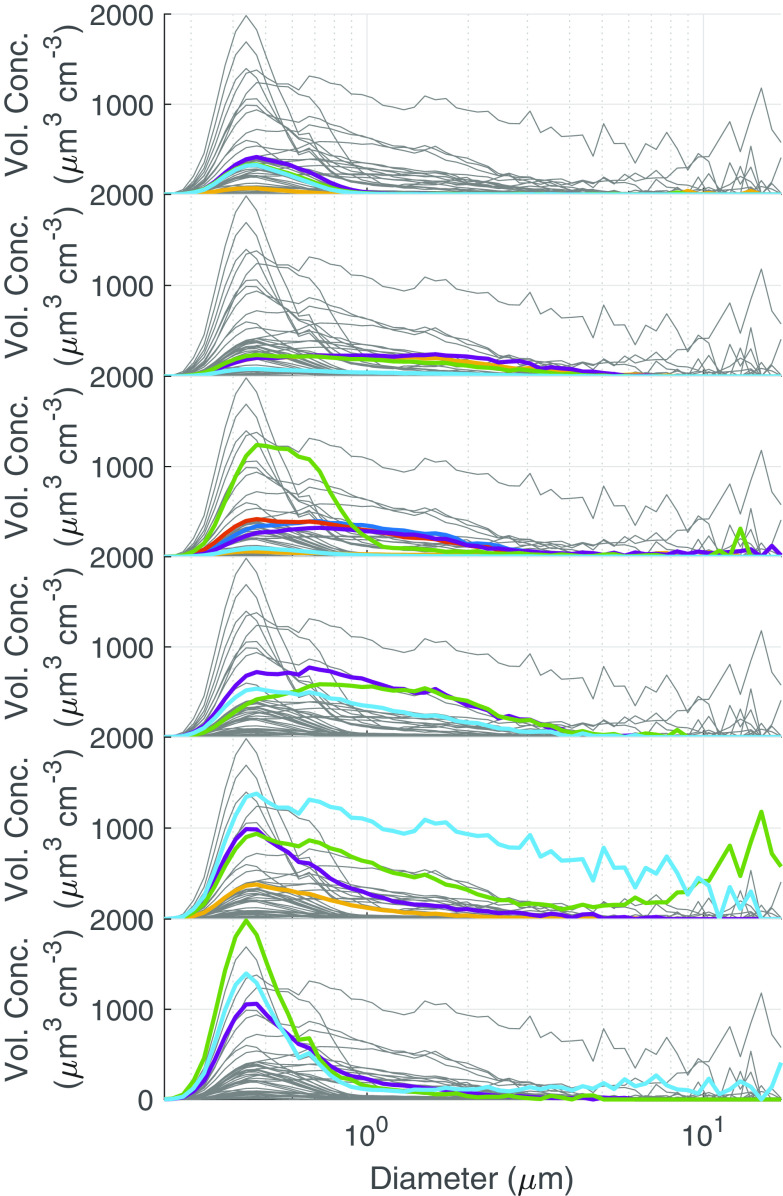
Volumetric size distribution of BSA added as a supplement to SM buffer at various concentrations. The gray traces correspond to all data aligned on the same graph for reference. The colored traces are replicates corresponding to the indicated BSA concentration in each panel. The panels from top to bottom are: 0.01% BSA, 0.1% BSA, 1% BSA, 2.5% BSA, 5% BSA, and 10% BSA.

**FIG 6:**
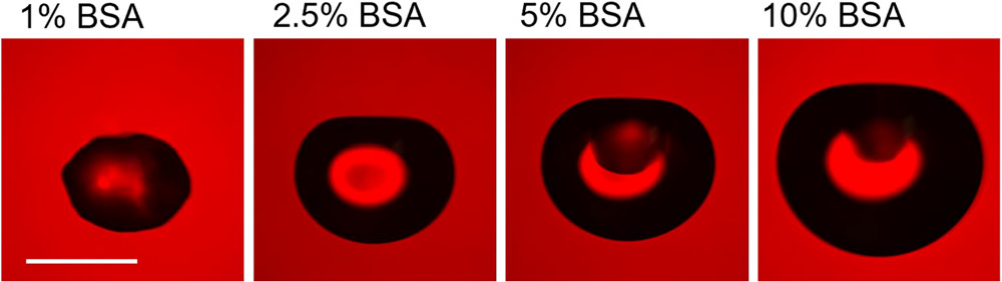
Evaporated particles levitated in an acoustic trap. In each case, the initial droplet volume was ~5 μL. Images shown were collected 2 h after initial trapping. For 0.1% BSA, droplets evaporated to a size that was too small to remain trapped. Bar, 500 μm.

To address whether the nebulizing medium is affecting the size of aerosol particles, we investigated average particle sizing data recorded from a high-resolution aerosol spectrometer monitoring the phi6 experiments. Overall, most of the aerosol particles generated in the testbed were fairly monodisperse and submicrometer-sized for each nebulizer medium. For the higher-performing nebulizing medium (1 to 10% BSA/SM), there was a small increase in the number of larger particles, which is especially apparent above 1 μm (Fig. 4). For additional clarity, the number distribution plots were converted to volume concentration, assuming spherical droplets (Fig. 5). The data indicate a clear trend in the larger particulate size as BSA concentration increased. However, at 10% BSA, there is a sudden decrease in the large particle size concentration. These data indicate that BSA supplementation reduces the extent of particle evaporation in the biological aerosol exposure testbed (BAET). The simplest explanation for the apparent decreased particulate size for the 10% BSA data is that the reduced extent of evaporation leads to much larger particles in the BAET and gravitational settling is preventing their detection. To demonstrate the effect of BSA concentration on particle size, acoustically levitated droplets were desiccated in open laboratory air. As shown in Fig. 6, for the same initial droplet volume (~5 μL), the size of the dried particles increases with increasing BSA concentration, consistent with the size trends observed with the Collison nebulizer. This trend can be attributed to the higher percentage of nonvolatile solutes and hindered water evaporation due to the formation of a viscous protein shell (20). The evaporation trend with respect to BSA content will remain consistent at smaller aerosol sizes. Taken together, these data indicate that at least some of the protective effect of BSA can be attributed to reducing exposure of the phage to the air-water interface by reducing the extent of particle evaporation.

## DISCUSSION

Given the SARS-CoV-2 pandemic, the question of whether infectious airborne viruses persist in high-traffic hubs remains a great concern and will require continued investigation. As developed above, the safety and tractability of phages for such studies offer a bounty for investigators interested in studying viral spread and aerosol mitigation strategies. Until recently, the SARS-CoV-2 surrogate phi6 was shown to have a low survival rate in droplets (13) or aerosols (10, 17) when viral suspensions were prepared in standard buffers or water. These findings likely limited the utility of phi6 to serve as an enveloped virus surrogate for applied aerobiology, especially when the goal is to mimic a highly transmissible airborne virus like SARS-CoV-2.

### Is total dissolved protein the key protective component in respiratory aerosols?

A recent study showed significant improvement in the recovery of dried phi6 droplets when suspensions were prepared in human saliva (13). Others have suggested that saliva serves as an organic barrier against environmental extremes (21); however, the specific constituents within saliva that confer the protective effect are unknown (13). Respiratory aerosols have been shown to be primarily composed of organic constituents, which affects droplet hygroscopicity (22). The composition of liquid suspensions used to generate aerosols (nebulizing media) has been shown to strongly affect the survival of airborne viruses (10, 15, 23, 24). Improvements in recovery were detected when researchers supplemented nebulizing medium with allantoic fluid (10) or cell culture medium (15), suggesting that the amount of dissolved protein is protective to airborne viruses. Furthermore, studies focused on the survival of viruses in (nonaerosolized) droplets have demonstrated protective effects for BSA supplementation (16, 25). These findings motivated us to address the question of whether protein supplementation will confer protective effects to airborne surrogate viruses phi6, phiX174, and MS2 and nonsurrogate tailed phage 80α. Supplementation of BSA into SM buffer showed significant protective effects to phi6, MS2, and 80α, but no significant improvements in recovery were detected for phiX174. The improvements were not monotonically dose-dependent for protein supplementation, which is consistent with what others have reported before (15, 26, 27). We identified combinations of BSA/SM that outperformed a commercially formulated artificial saliva for phi6, MS2, and 80α. Mucin supplementation significantly improved survival of most phages in our testbed, in agreement with what was previously shown for MS2 (15). Taken together, the data above suggest that the key protective feature in saliva is total dissolved protein content. From our searches, protein is among the constituents suspected to confer survivability on respiratory viruses (13), but this has not been directly tested. In principle, the role of proteins in saliva could be directly probed if methods (e.g., affinity purification) were used to remove the most abundant proteins from saliva without disturbing the sample matrix.

Recently, investigators have used microcopy methods to understand the distribution of viruses and protein supplements in droplets. Using a lipid-labeling fluorescent dye, phi6-containing samples showed a disperse labeling throughout the droplet and labeled phi6 were not associated with large mucin aggregates (28). The distribution pattern of mucin changed as relative humidity was decreased. Initially, mucin formed an apparent shell-like structure on the surface of the droplet. Similar features have been observed for MS2; scanning electron microscopy (SEM) images suggest that mucin forms a protective cross-linking that encapsulates viruses in aerosols (26). We concur that the simplest explanation for the increased survivability is that proteins provide a protective encapsulation around the virus that protects it from desiccation by reducing the extent of evaporation. Further investigation of this effect and why protein supplementation does not result in dose-dependent protection (27) will likely require superresolution immunofluorescent microscopy methods to probe the distribution of key features in droplets.

### Nebulizing medium that protects airborne viruses.

In this report we have identified common and inexpensive lab reagents that confer increased aerosol survivability on phi6 and other phages. These findings will be useful for applications in which researchers wish to improve the survivability of these (and likely other) aerosolized viruses to better approximate highly transmissible airborne viruses like SARS-CoV-2. Moreover, our data suggest a degree of tunability to the level of protection, depending on the amount of protein supplementation or the presence of buffer/ionic solvent. The tunability feature may be useful if there is concern that artificial nebulizing medium outperforms natural suspensions, which has been previously reported for MS2 (15). In our view, a key next step is to determine the duration of the protective effect of our viability preservation medium in static aerosol experiments. Additionally, there is a need to move from descriptive studies, such as the current work, to mechanistic investigations that focus on understanding key structural and genetic features that confer stability on viruses in aerosols. These data would be useful for determining the variables that contribute to the persistence of viruses in aerosols.

## MATERIALS AND METHODS

### Bacteria and phages.

phi6 and host Pseudomonas syringae were kindly provided by Krista Wigginton (University of Michigan). MS2 and phiX174 and their cognate Escherichia coli host strains were obtained from ATCC (15597 and 13706, respectively). Staphylococcus aureus RN4220 and RN0027 were obtained from BEI Resources. Phage 80α was isolated from RN0027 by mitomycin C induction and propagated on RN4220. All cultures were propagated on LB broth and LB agar (Teknova; catalog no. L1110) at 37°C or 26°C (P. syringae only). Overnight cultures were prepared from single colonies inoculated into 4 mL LB in 16-mm borosilicate glass test tubes with vented caps and incubated with aeration overnight.

### Phage stock maintenance.

High-titer phage lysates were produced using a standard full plate titer plaque assay by mixing ~1 × 10^6^ PFU with 100 to 200 μL of overnight culture, inoculated into molten “top” LB agar (0.6% agar, supplemented with 5 mM CaCl_2_ and 10 mM MgCl_2_), and plated onto LB agar plates. After overnight incubation, 5 mL SM buffer (Teknova catalog no. S0249; 10 mM Tris-HCl, 100 mM NaCl, 50 mM MgSO_4_, 0.01% gelatin, pH 7.5) was added to confluent plates and agitated by a rotary shaker at room temperature for ~1 h. The SM/top agar layer was scraped into 50-mL conical tubes, vortexed for 10 to 15 s, and then centrifuged (~30 min, ~5,000 × *g*, 4°C) to separate phage from cells and agar. The resulting supernatant was filter sterilized, and then titers were determined using standard phage titer techniques, described below.

### Phage quantification.

Bacterial lawns were formed by mixing 100 μL (for E. coli C-3000 and S. aureus) or 200 μL (P. syringae), of overnight culture with 4 mL of 0.6% molten top agar and immediately plating on an LB plate. phiX174 plating methods are described below.

Phage titer determination was performed using the standard double layer agar method. Typically, 100 μL of phage suspensions was serially diluted in 900 μL of SM buffer, and 10 μL of the dilutions was spotted on the lawn in triplicates. After overnight incubation at the temperatures indicated above, single plaques from the dilution containing the highest number of countable plaques were enumerated.

The inherent large plaque size produced by phiX174 required the following modifications to reduce plaque size for accurate quantification: 500 μL of overnight inoculum was injected into 1% top agar to make the bacterial lawn for each plaque assay. Additionally, plaques were read after 3 h of incubation at 42°C.

### Viability preservation nebulizing medium.

High-titer lysates were diluted 1,000× in each test medium. Bovine serum albumin (BSA; Sigma-Aldrich A9418-500G bioreagent; A2153-500G) was added as a supplement at 0.01%, 0.1%, 1.0%, 2.5%, 5.0%, and 10% (wt/vol) concentrations of BSA in SM buffer. Artificial saliva (Pickering Lab 1700-0317 and 1700-0316; ASTM E2720-16, pH 7) was prepared with or without 0.3% type II mucin from porcine stomach, according to the manufacturer’s recommendations.

### Aerosolization of phi6.

The biological aerosol exposure testbed (BAET, Fig. 1) is a self-contained and controlled aerosolization system utilizing a CH Technologies, Inc. (Westwood, NJ) Collison nebulizer with a Jet-6 configuration (model CN25). The nebulizer produced aerosols with a mean diameter of approximately 2 μm (wet size, before drier) at a constant airflow rate of 12.5 liters per minute (LPM).

For each experiment, the Collison nebulizer was filled with 25 mL of phage prepared in test medium as described above. The component mixtures and control were dispersed individually with the nebulizer within the BAET for 10 min into a mixing chamber (3 in. in diameter and approximately 14.25 in. in length) and TSI, Inc. (Shoreview, MN) diffusion drier (model 3062). This was done to establish an equilibrium concentration before the exposures were conducted. During this time, a temperature and relative humidity (RH) measurement was taken. Particle sizing was measured using a Welas Promo 2000 (Karlsruhe, Germany), a white-light scattering aerosol spectrometer system that measures both particle size distribution and concentration (numbers/square centimeter). The temperature/RH and Promo samples were taken at the 9-min mark for approximately 1 min. After the 10-minute stabilization period, the aerosol flow was isolated from the temperature/RH probe and Promo, and an Ace Glass Incorporated air sampling impinger (AGI-30) was connected to the BAET (Fig. 1A). The AGI-30 was filled with 20 mL of sterile SM buffer and operated at a flow rate of 12.5 L/min. The AGI-30 sampled the bioaerosol from the BAET for approximately 5 min for each test medium. After each spray, samples were withdrawn from the AGI-30 and nebulizer, and titers were determined on the same day.

To further explore the effect of BSA concentration on particle size, droplets of test medium were levitated in an acoustic trap. The design of the acoustic trap (29) and its use to study the evaporation of model respiratory compounds (30) are described elsewhere. Here, droplets (~5-μL initial volume) were generated using a syringe and a blunt-end needle and were levitated in open laboratory air (RH, <40%), and the evaporation process was tracked by brightfield imaging.

### Data analysis.

We performed four (six for phi6) biological replicates for each test medium, each with three technical replicates. The titers of the sample loaded into the nebulizer were determined after each spray.

An initial series of experiments found no detectable difference between the titers of samples in the nebulizer at the beginning of the spray session and those at the end of the spray. Therefore, the titer of samples after the spray (designated input titer in equation 1) was used in equation 1 to calculate an average titer baseline for the maximum number of viruses that could be transferred assuming no losses. Equation 2 was applied to divide the titer of replicates by the average theoretical maximum titer. The viability ratio from equation 2 was used to calculate an average and standard deviation for each data set when biological replicates were combined.

(1) $$\text{theoretical maximum titer}=\frac{\text{input titer}\times \;\;\text{volume aerosolized (0}.25 mL)}{\text{AGI collection volume (20 mL)}}$$

(2) $$\%\;\text{recovery}=(\frac{\text{titer of aerosolized}\;(\text{AGI})\;\text{phi6 for each medium}}{\text{theoretical maximum titer based on averaged known inputs}})\;\times 100\%$$

Number concentration plots, based on the total number of particulates per unit volume, were converted into volume density assuming spherical particles according to the following equation:

$$u_{v}=\frac{4}{3}\pi \overset{\infty }{\underset{0}{\int }}r^{3}n\left(r\right)dr$$

where *u*(*v*) is volume distribution, *r* is the radius, and *n*(*r*) is the number density distribution.
